# Primary Cutaneous Nocardiosis: A Rare Presentation of Nocardiosis

**DOI:** 10.7759/cureus.5860

**Published:** 2019-10-07

**Authors:** Ezza Fatima Tariq, Muhammad M Anwar, Usman A Khan

**Affiliations:** 1 Nephrology, Oklahoma University Health Sciences Center, Oklahoma City, USA; 2 Biochemistry, King Edward Medical University (KEMU) / Mayo Hospital, Lahore, PAK; 3 Internal Medicine and Nephrology, University of Oklahoma Health Sciences Center, Oklahoma City, USA

**Keywords:** nocardia, hand nocardiosis, cutaneous nocardiosis, acid-fast bacilli

## Abstract

Nocardia is an uncommon gram-positive, weakly acid-fast bacterium that causes systemic or localized suppurative disease in humans and animals. Nocardiosis is typically regarded as an opportunistic infection, but approximately one-third of the patients are immunocompetent. The most common presentation is pulmonary disease (39%) followed by systemic involvement, defined as involvement of more than two sites; cutaneous presentation constitutes only 8% of the cases. Nocardia is widely distributed geographically; however, in the US, it is mostly found in warm and dry areas of South West and South East.

We present a perfect case of cutaneous nocardiosis of a 70-year-old male, who presented with a traumatic splinter injury, leading to pustules formation on the right index finger, along with erythema and induration of the right arm. The patient was empirically diagnosed and treated for cellulitis, with amoxicillin and clavulanic acid, resulting in deterioration of the wound. The patient underwent incision and drainage and wound culture grew nocardia.

The index of suspicion should be kept in mind while treating infectious blisters which have failed outpatient cellulitis treatment, immunocompromised hosts, and in nocardia prevalent regions.

## Introduction

Nocardia is an aerobic gram-negative, acid-fast filamentous bacillus. It is known to have 80 species, with 33 known to cause disease in humans [1-3]. Nocardia is primarily known to cause diseases in immune-compromised patients such as organ transplant recipients, HIV-AIDS patients and people taking long-term corticosteroid therapy. The most common manifestation is pulmonary disease; however, disseminated disease or central nervous system (CNS) involvement is also seen in severely immune-compromised patients. Some cases are also seen in immune-competent patients with the most common manifestation being the cutaneous disease.

The incidence of nocardiosis in the early 1970s was recorded to be 500-1000 cases per year in the United States [4]. However, the incidence now is approximated to be higher. Among these cases, cutaneous nocardiosis is extremely rare, accounting for only 8% of the cases. Moreover, it cannot be reliably diagnosed based on the clinical picture alone. Therefore, a high index of suspicion should be kept. We present a case of hand cutaneous nocardiosis after a splinter injury in an immune-competent patient who was successfully diagnosed and treated.

## Case presentation

We present a case of 70-year-old male with a past medical history of chronic obstructive pulmonary disease (COPD) and tobacco use who presented in the ED with a complaint of a skin lesion on the right thumb with swelling and pus drainage. He also had erythema, induration, and red streaks along the lymphatic vessels in the right arm. The patient reported fever and chills with no other associated signs or symptoms.

The patient also reported that he remembered being scratched by his pet cat and had an incident of splinter injury by a wood twig a few weeks prior to his presentation. The patient lived in poor living conditions as well.

Initial diagnosis of cellulitis was made, and amoxicillin/clavulanic acid was prescribed. However, this resulted in the swelling and induration to exacerbate which caused the patient to seek medical attention again, two days later.

**Figure 1:**
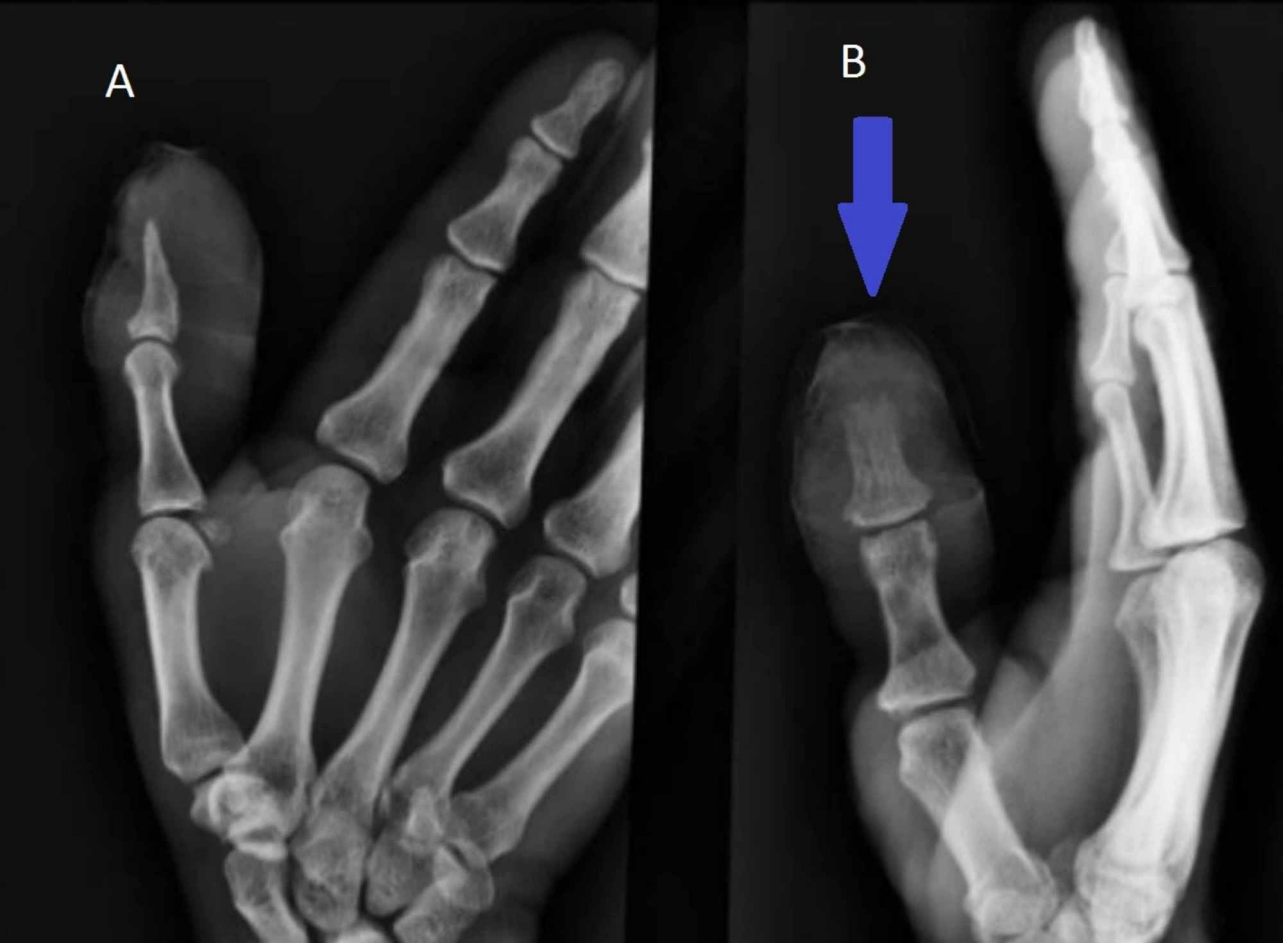
The CT scan of right hand. (A) Antero-posterior view, (B) lateral view. Blue arrow shows soft tissue swelling and osteomyelitis of distal phalanx of the thumb.

On examination, there was a lesion on his right thumb, with red streaks in the forearm (Figure 1). No associated lymphadenopathy was seen. Incision and drainage procedure was performed and drainage was sent for culture. Following incision and drainage, the swelling and induration subsided.

Upon investigation, the white blood cell (WBC) count was found to be 7.4 x 10^3^/mm^3^ and erythrocyte sedimentation rate (ESR) was 46 mm/h. The culture of drainage showed aerobic actinomycetes, most likely nocardia. CT scan of the right hand (Figure 1B) showed osteomyelitis of distal phalanx of the thumb and soft tissue swelling around it. These findings are indicative of the spread of infection to the bone.

The diagnosis of cutaneous nocardiosis was made and the patient was successfully treated with ceftriaxone and sulfamethoxazole/trimethoprim.

## Discussion

Nocardia is a gram-positive, acid-fast bacteria and is classified under the family of Nocardiaceae, order Actinomycetales, suborder Corynebacterineae. The species which is most commonly associated with cutaneous nocardiosis is N. brasiliense (80% of the cases) [1, 5-7].

The nocardia species is a saprophyte and is found in a vast variety of places in the environment, including freshwater, saltwater, soil and decomposing waste of plant and animal origin. In the US, it is demographically found mostly in the warm and dry areas of the South West and South East [1,5,8,9].

Nocardiosis is a disease most commonly manifesting in immune-compromised patients. However, some cases of immune-competent patients have also been reported.

The clinical manifestations of the nocardia infection include skin (8%), lung (39%) involvement or the disseminated (32%) picture. It can also spread and involve the CNS [10,11]. The cutaneous disease may involve superficial skin alone, show lympho-cutaneous involvement, disseminate mycetomas or sporotrichoid presentation [2,4]. The superficial disease may present as cellulitis or pustular disease. The lympho-cutaneous presentation has skin and lymph nodes involvement and therefore can be confused with tuberculosis. Mycetomas present with a triad of tumefaction, draining sinus and presence of granules in discharging pus. Sporotrichoid presentation is often misdiagnosed to be fungal in origin [1].

Primary cutaneous disease is often followed by traumatic implantation of the nocardia bacterium after splinter injury [5,6,12]. The secondary cutaneous disease can occur after the dissemination of the pulmonary disease leading to secondary focus created in the skin.

We present a unique case of cutaneous nocardiosis in which the patient presented with a pustular lesion, erythema, and induration and had a history of splinter injury a few weeks prior. As the clinical picture was very similar to cellulitis, which is in most settings caused by Staph. Areus or Streptococcus species, amoxicillin/clavulanic acid was prescribed [5]. However, aggravation of swelling and induration lead to an investigation for other causative agents. Culture of drained fluid, collected after incision and drainage, showed growth of aerobic actinomycetes, diagnosis of nocardia was made and TMP/SMX treatment was prescribed which lead to a successful recovery.

Our case is unique because cutaneous nocardiosis is a rare manifestation and has a tendency to be misdiagnosed because it can mimic other skin diseases such as pyoderma, Leishmaniasis, tuberculosis (when it presents as a lympho-cutaneous disease) and cellulitis (such as in this case) [5-6]. This case is also distinctive due to the fact that cutaneous nocardiosis is a rare disease itself and should be suspected especially in cases of traumatic injury or splinter injury, as was seen in the case presented.

Clinical diagnosis may be not very reliable. Smear by gram staining is done in most cases. Gram staining shows thin filaments of gram-positive aerobes. Ziehl-Neelsen stain with 1% sulfuric acid is used instead of 20% for the identification of Nocardia species, however, it is less sensitive [5-6]. Nocardia grows on most media, culture takes 5-7 days to grow. Other procedures include biochemical DNA probing, DNA sequencing, PCR-RFLP, ribotyping PCR [1,5,8,9]. These techniques can help identify nocardia to species level which gives important information about drug susceptibility. However, they are not routinely used in the clinical setup.

Despite the fact that most species are susceptible to TMP/SMX, other drugs might be used in the case of sulfonamide allergy or resistance. Alternatives to TMP/SMX in such cases include amikacin, minocycline, imipenem, and third-generation cephalosporins [5,10,11].

## Conclusions

Cutaneous nocardiosis is a rare presentation. There is a high chance to confuse it with other skin conditions, as in the case presented the first impression made was of cellulitis. Therefore, we suggest that physicians should anticipate the probability of nocardia as the causative agent in cases that arise in the nocardia endemic region or those cases initially not responsive to the empirical treatment. Possibility of nocardia infection should also be suspected in patients with a history of traumatic inoculation.
